# Retraction Note: microRNA-17 functions as an oncogene by downregulating Smad3 expression in hepatocellular carcinoma

**DOI:** 10.1038/s41419-026-08829-4

**Published:** 2026-04-29

**Authors:** Zhufeng Lu, Xiuhua Li, Yongfeng Xu, Miaomiao Chen, Wei Chen, Tao Chen, Qinghe Tang, Zhiying He

**Affiliations:** 1 Department of Anesthesia, Zhongshan Hospital, Fudan University, Shanghai, 200032 P. R. China; 2 Institute for Regenerative Medicine, Shanghai East Hospital, School of Life Sciences and Technology, Tongji University, Shanghai, 200123 P. R. China; 3 Liver Cancer Institute, Zhongshan Hospital, Fudan University, Shanghai, 200032 P. R. China; 4 Department of Hepatobiliary Surgery, Sun Yat-sen Memorial Hospital, Sun Yat-sen University, 33 Ying Feng Road, Guangzhou, Guangdong 510289 P. R. China; 5 Department of Hepatobiliary and Pancreatic Surgery, Shanghai East Hospital, Tongji University, Shanghai, 200123 P. R. China

Retraction Note to: *Cell Death & Disease* 10.1038/s41419-019-1960-z, published online 26 September 2019

The Editors-in-Chief have retracted this article. An investigation conducted after its publication discovered that the *p21* band shown in Figure 5D appears to overlap with a portion of the *c-Myc* band in Figure 4B in [1] after a horizontal flip. The authors informed the Journal that the source data for this research is no longer available. The Editors-in-Chief therefore no longer have confidence in the integrity of the research presented in this article.

The authors did not explicitly state whether they agree or disagree with this retraction.
